# Facile strategy for the fabrication of noble metal/ZnS composites with enhanced photocatalytic activities†

**DOI:** 10.1039/c9ra07163f

**Published:** 2020-01-27

**Authors:** Xuekun Jin, Fengjuan Chen, Dianzeng Jia, Yali Cao, Haiming Duan, Mengqiu Long

**Affiliations:** Key Laboratory of Energy Materials Chemistry, Ministry of Education, Key Laboratory of Advanced Functional Materials, Autonomous Region, Institute of Applied Chemistry, Xinjiang University Urumqi Xinjiang 830046 China jdz0991@gmail.com; School of Physics Science and Technology, Xinjiang University Urumqi 830046 Xinjiang PR China cfj2008meet@163.com; Hunan Key Laboratory of Super Micro-structure and Ultrafast Process, Central South University Changsha 410083 China

## Abstract

The introduction of noble metal nanoparticles to photocatalysts can effectively improve the separation efficiency of the photogenerated electron–holes. Therefore, noble metal/ZnS composites were synthesized using a low-temperature solid-phase chemical method with sodium borohydride as the reducing agent. The characterization results showed that the noble metal/ZnS composites have been successfully obtained and that the noble metals were distributed on the surface of ZnS. The catalytic results suggested that the composites exhibited improved activity after introduction of noble metals, which can be attributed to the rapid migration of carriers and the enhancement of the light absorption, mainly owing to the tight combination between the ZnS and noble metals and the plasmon resonance effect of the noble metals. The catalytic mechanism was explored by using photoluminescence spectroscopy, photocurrent spectra, valence band X-ray photoelectron spectroscopy (XPS-VB) spectra and capture agent experiments, and a possible mechanism was proposed. This work provides a new strategy for the high-volume synthesis of noble metal-based composite photocatalysts, which could be helpful for sustainable development.

## Abstract 2

The introduction of noble metal nanoparticles to photocatalysts can effectively improve the separation efficiency of the photogenerated electron–holes.

## Graphical abstract

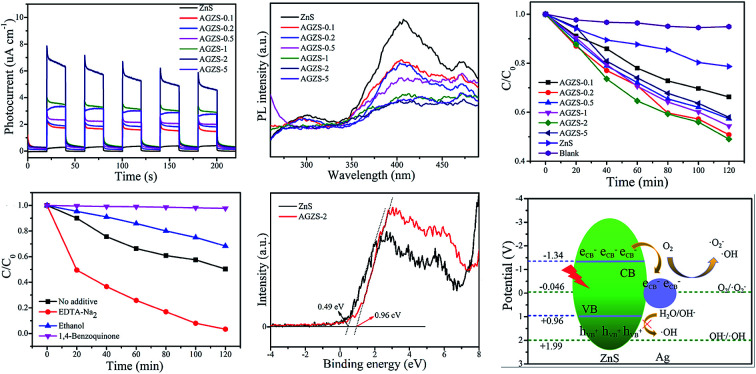

## 1. Introduction

With the fast development of the economy, environmental problems have become increasingly severe. Therefore, people have adopted many ways to solve this problem, and photocatalytic technology has received extensive attention owing to its unique advantages.^1–3^ As it is well known, photocatalytic materials are at the core of the technology. After decades of research, a series of semiconductor-based photocatalysts have been explored.^4–8^ Among them, ZnS has received significant attention owing to the high efficiency and low price. However, fast recombination of photo-generated carriers, results in a decrease in the separation ability, and leads to a reduced catalytic activity. Hence, a novel approach is urgently needed to obtain high-activity photocatalysts with a higher carrier separation efficiency.

It has been found that the catalytic activity of ZnS can be improved by means of depositing noble metals, compounding semiconductors, exploring new materials, and so forth.^9–13^ Among these techniques, depositing noble metals is an effective approach to enhance the activity. This is because noble metal nanoparticles have a good electrical conductivity and electron-trapping ability, and can act as an electron medium to generate resonance in an electric field caused by illumination, which is called the surface plasmon resonance effect (SPR), and the effect can promote effective separation of the electron–hole.^14–18^ For example, Ayodhya *et al.* synthesized Zn_*x*_Ag_1−*x*_S composites using the hydrothermal method, and the photocatalytic results showed that the composites exhibited an excellent photocatalytic degradation activity to organophosphorus pesticides after the introduction of Ag.^19^ The enhanced performance may be due to the plasmon resonance effect which causes the photogenerated electrons on the ZnS conduction band to be injected into the Ag surface, thereby promoting the efficient separation of the photogenerated carriers.^20^ Thus, depositing noble metals has been considered to be an effective strategy to enhance the photocatalytic properties.

In general, the composition, structure and properties of materials are closely related to their catalytic properties. It has been found that there are many methods for fabricating noble metal/sulfide composites, such as the microwave assisted reduction method, the photoreduction coupled water bath method, and the *in situ* deposition method.^21–26^ However, the above mentioned methods have some disadvantages, such as a complicated operation, high cost, and can be difficult to control. Therefore, it is highly desirable to find a simple and fast technique to obtain noble metal/sulfide composites. At present, our group has synthesized a series of nanomaterials with excellent photoelectric properties by using the low-temperature solid-phase chemical method.^27–30^ However, through consulting numerous studies, it has been found that the synthesis of noble metals/sulfides composites using this method have not been reported so far.

In this paper, Ag/ZnS composites and Au/ZnS composites were synthesized using a low-temperature solid-phase chemical method with the aid of the reducing agent sodium borohydride (NaBH_4_). The photocatalytic activity of the composites was studied by degrading methyl orange (MO) under UV light, and the photocatalytic mechanism was further discussed. It was found that the noble metal nanoparticles in the composites synthesized using the method have strong binding with ZnS, which facilitates the rapid separation of the photogenerated carriers and improves the catalytic activity.

## 2. Experimental section

### 2.1 Preparation of the noble metal/ZnS composites

Ag/ZnS composites were synthesized using a low-temperature solid-phase chemical method. First, Zn(CH_3_COO)_2_ 2H_2_O (0.01 mol) was added to the agate mortar. After grinding for 10 min, NH_2_CSCH_3_ (0.01 mol) was added. After grinding for 40 min, NaBH_4_ (0.05 mol) was added and the mixture was continually milled for 40 min. Subsequently, AgCH_3_COO (0.0015 g) was added and milled for 60 min to obtain the product. Finally, the obtained product was washed several times with deionized water and ethanol, and dried for 10 h at 80 °C to obtain the Ag/ZnS composites AGZS-0.1. Here, when the mass percentage of Ag and ZnS in the final product is 0.1, the composites are named AGZS-0.1. In a similar manner, composites AGZS-0.2, AGZS-0.5, AGZS-1, AGZS-2, and AGZS-5 were also obtained with different compositions.

In order to further study the influence of noble metals on the composition, structure and performance of ZnS, Au/ZnS composites were also synthesized under the same conditions as those described above. The difference was that AgCH_3_COO (0.0015 g) was replaced with AuCl·HCl·4H_2_O (0.021 g, the mass percentage of Au and ZnS was 0.1), and the Au/ZnS composite was obtained and designed as AUZS-0.1. By changing the mass percentage of Au and ZnS, composites AUZS-0.2, AUZS-0.5, AUZS-1, AUZS-2 and AUZS-5 with different compositions were also prepared.

For the convenience of comparison, pure ZnS was also synthesized without NaBH_4_, AgCH_3_COO and AuCl·HCl·4H_2_O.

### 2.2 Characterization

The crystal structure of the composites was analyzed using an X-ray powder diffractometer (D8 Advance, Bruker). The microscopic morphology of the composites was characterized by field emission transmission electron microscopy (JEOL, JEM2011F). The specific surface areas were analyzed using the Brunauer–Emmett–Teller (BET) method on a Micromeritics ASAP 2460 apparatus. The elemental composition of the composites was analyzed using X-ray photoelectron spectroscopy (XPS, SCIENTIFIC ESCALAB 250Xi, Thermo Scientific). The ultraviolet-visible absorption spectrum of the composites was measured using an UV-visible spectrometer (H-3900, Hitachi). The photoluminescence spectra of the composites were measured using a photoluminescence spectrometer (F-4500, Hitachi). The photocurrent of the composites was tested on an electrochemical workstation (CHI 660B, Shanghai Chenhua). The catalytic performance of the composites was analyzed on a photochemical reactor (XPA-7, Nanjing Hanjiang Power Plant).

### 2.3 Photocatalytic tests

The catalytic performance of the composites was performed on an XPS reactor with a 300 W mercury lamp source. As the energy of the light source is high, more heat is generated, and it is necessary to continuously pass condensed water through for circulated cooling. The catalytic performances of the composites were assessed using MO as a model.

The procedure was performed as follows: 25 mg catalyst was added to 50 mL MO solution (10 mg L^−1^). In order to reach the adsorption equilibrium between the catalyst and MO solution, the adsorption was allowed to progress for 2 h under dark conditions. Subsequently, the light source was turned on for testing. 3.5 mL of the reaction solution was taken at intervals of 20 min, centrifuged at a certain speed (rotation speed of 10 000 rpm) for 10 min, and the supernatant was taken and analyzed for the concentration of MO. The concentration of MO was measured using an UV-vis spectrometer with a range of 300–700 nm.

In order to study the active substances in the catalytic process, a capture agent experiment was carried out. The capture agents adopted were EDTA-Na_2_, ethanol and 1,4-benzoquinone. The experimental procedure was the same as that described above, except that a certain amount of capture agent was added to the system.

## 3. Result and discussion

### 3.1 Characterization of the Ag/ZnS composites

**Fig. 1:**
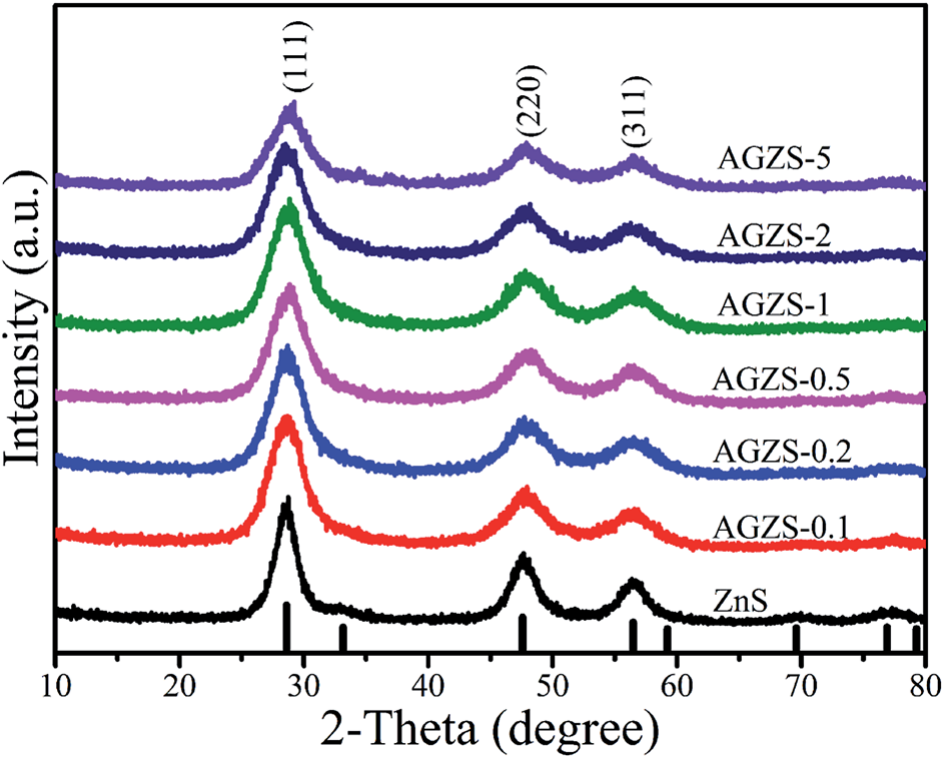
XRD patterns of pure ZnS and Ag/ZnS composites.

Fig. 1 exhibits the X-ray diffraction (XRD) patterns of the Ag/ZnS composites prepared by the low-temperature solid-phase reaction in the presence of NaBH_4_ and varying amounts of silver acetate. From Fig. 1, it can be noted that the diffraction peaks of pure ZnS are consistent with the standard card of cubic ZnS (JCPDS no. 05-0566).^31^ At the same time, the characteristic peaks of the Ag/ZnS composites are in agreement with that of pure ZnS, indicating that introduction of Ag does not change the crystal structure of the composites. No characteristic peak for Ag was observed in the Ag/ZnS composites, which may be due to its low content. It is worth noting that the intensity of the diffraction peak corresponding to the (111) crystal plane of ZnS in Ag/ZnS composites gradually decreases with the increased amount of Ag, owing to the high dispersion of Ag, indicating that the amount of Ag had an important influence on the crystallinity.^32^

**Fig. 2:**
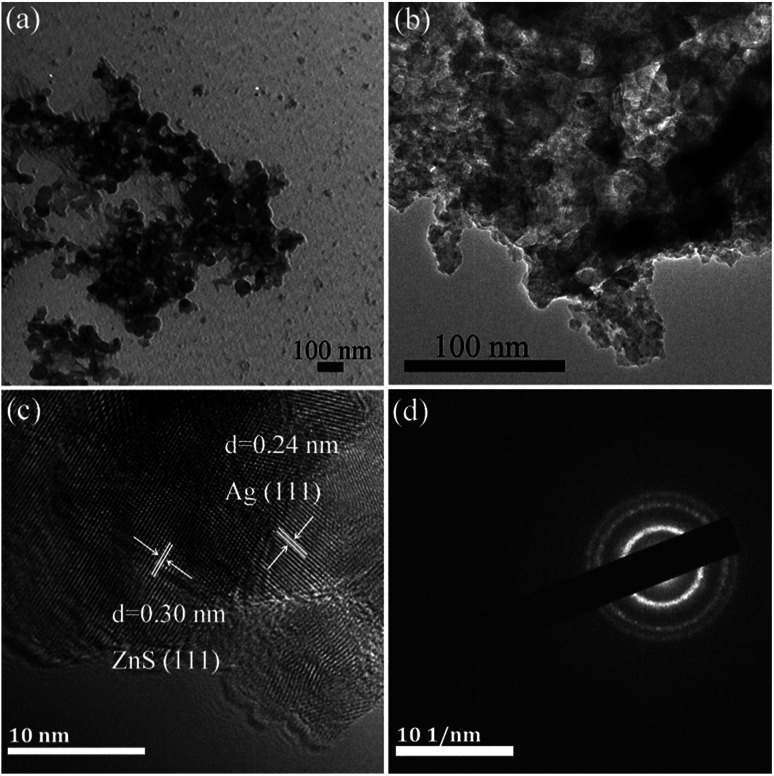
TEM images of ZnS (a), Ag/ZnS composite AGZS-2 (b); HRTEM image (c), and SAED patterns of the composites AGZS-2 (d).

In order to clearly observe the morphology of the samples, transmission electron microscopy (TEM) analyses were conducted as shown in Fig. 2. It can be seen that the ZnS nanoparticle with a size of about 50 nm, has a slight agglomeration (Fig. 2a). In Fig. 2b, as for the composites AGZS-2, a small amount of Ag nanoparticles are present on the surface of ZnS. To further study the structure of the composites, high-resolution transmission electron microscopy (HRTEM) analysis was performed. In Fig. 2c, the composite AGZS-2 has clear lattice fringes, in which the lattice spacing of 0.30 nm corresponds to the (111) crystal plane of cubic ZnS, and the lattice spacing of 0.24 nm is consistent with the (111) crystal plane of the cubic Ag, suggesting the presence of the ZnS and Ag phases in the region.^33^ At the same time, there is a clear boundary between the ZnS and Ag lattices, indicating the formation of Ag/ZnS composites. In addition, the SAED results (Fig. 2d) indicate that the composites have a polycrystalline property.

Specific surface areas on the Ag/ZnS composites were also characterized using nitrogen (N_2_) adsorption–desorption. As shown in Fig. S1,† the Ag/ZnS composites all display a typical type IV isotherm with a distinct hysteresis loop. The BET specific surface area of the Ag/ZnS composites AGZS-0.1, AGZS-0.2, AGZS-0.5, AGZS-1, AGZS-2 and AGZS-5 are 38.85, 48.31, 60.04, 72.36, 239.83 and 53.01 m^2^ g^−1^, respectively. When the mass percentage of Ag to ZnS is two, the composite AGZS-2 exhibited the largest specific surface area in comparison with the others, which is helpful for the adsorption and carrier transport, and the composite AGZS-2 displayed the highest photocatalytic activity (as shown in Fig. 5a).

**Fig. 3:**
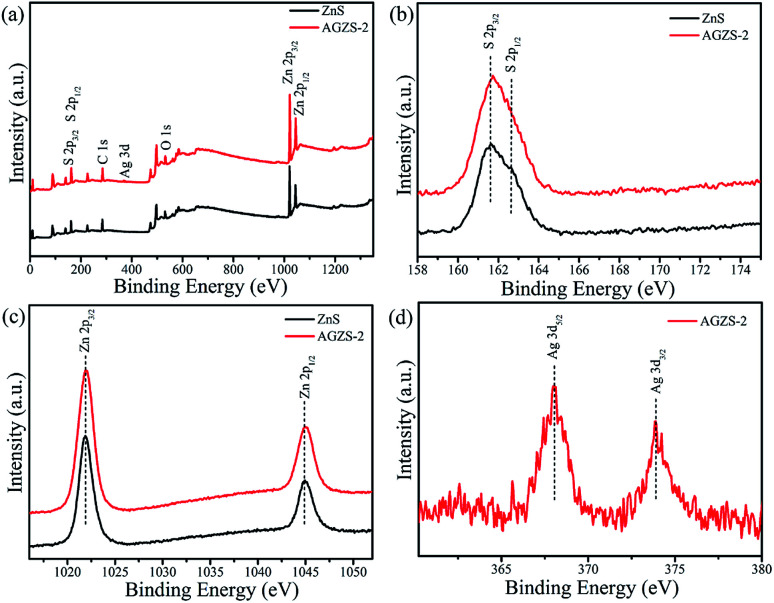
XPS spectra of the ZnS and Ag/ZnS composites AGZS-2 (a) survey; (b) S 2p; (c) Zn 2p; and (d) Ag 3d.

The chemical composition of pure ZnS and the composite AGZS-2 was studied using an XPS technique. In the survey spectra (Fig. 3a), characteristic peaks for S 2p, C 1s, Ag 3d, O 1s, and Zn 2p appear, in which C 1s and O 1s are derived from the test process,^34^ confirming the presence of the S and Zn elements for ZnS, and the presence of the S, Ag and Zn elements in the compositeAGZS-2. Pure ZnS exhibits characteristic peaks of S 2p_3/2_ and S 2p_1/2_ orbitals at 161.6 and 162.6 eV (Fig. 3b), respectively, indicating the existence of the S element.^35^ In contrast, the characteristic peaks of the S element in composite AGZS-2 were slightly red-shifted to 161.7 and 162.8 eV, respectively. In Fig. 3c, the pure ZnS exhibits characteristic peaks of Zn 2p_3/2_ and Zn 2p_1/2_ orbitals at 1021.9 and 1044.9 eV, respectively. However, there is a slight red shift for the Zn 2p characteristic peaks in the composites AGZS-2, suggesting that there are interactions between Ag and ZnS.^36^ The characteristic peaks at 368.0 and 373.9 eV in Fig. 3d correspond to the Ag 3d_5/2_ and Ag 3d_3/2_ orbitals of metallic Ag, respectively, indicating the presence of the Ag element in the composite AGZS-2.^37^ The above described results show that there is ZnS and Ag in the composites, that is, the Ag/ZnS composite photocatalyst is obtained. XPS analysis was further performed to measure the Ag percentage in the Ag/ZnS composites. The Ag percentage with respect to ZnS was found to be 1.5% in the composite AGZS-2, which further proved the expected Ag percentage in the ZnS.

**Fig. 4:**
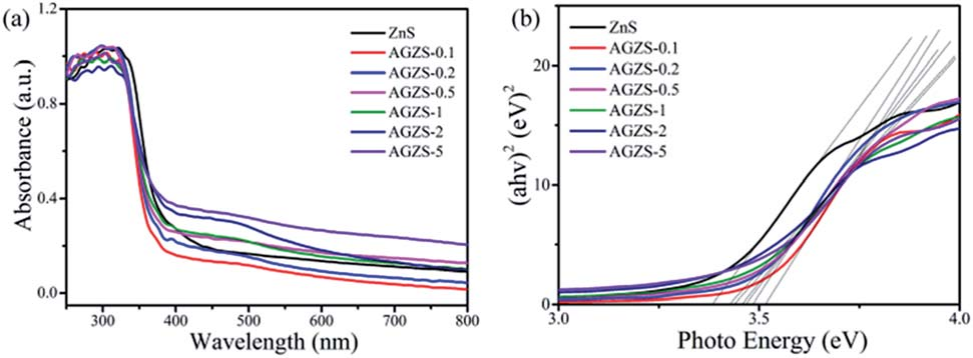
(a) UV-vis spectra; and (b) (*αhν*)^2^*versus hν* for the ZnS and Ag/ZnS composites.

The optical absorption properties of the ZnS and Ag/ZnS composites were studied as shown in Fig. 4. It can be found that pure ZnS has an absorption in the range of 250–400 nm, which is consistent with the literature.^38^ After introducing Ag, Ag/ZnS composites showed a slight blue shift, which may be due to its small size (Fig. 4a). At the same time, with the formation of Ag/ZnS composites, a new absorption band appeared in the range of 450–800 nm, which is caused by the SPR effect of Ag, further indicating the presence of Ag.^39^ As the amount of Ag increases, the absorption capacity increases continuously, indicating that the composites will exhibit improved photocatalytic activity.

According to the Tauc equation *αhν* = *A*(*hν* − *E*_g_)^*n*^,^40^ the band gap energy of the samples can be estimated by plotting the photon energy (*hν*) by (*αhν*)^1/2^, as shown in Fig. 4b. It can be seen that the band gap energies (*E*_g_) of ZnS, the Ag/ZnS composites AGZS-0.1, AGZS-0.2, AGZS-0.5, AGZS-1, AGZS-2, and AGZS-5 are 3.38, 3.52, 3.49, 3.47, 3.46, 3.43 and 3.44 eV, respectively. It can be seen that with the increase in the Ag content, the band gap of the Ag/ZnS composites decreases first and then increases. Furthermore, the *E*_g_ of composite AGZS-2 is the smallest, which may be related to its structure and properties.

### 3.2 Photocatalytic activity of the Ag/ZnS composites

**Fig. 5:**
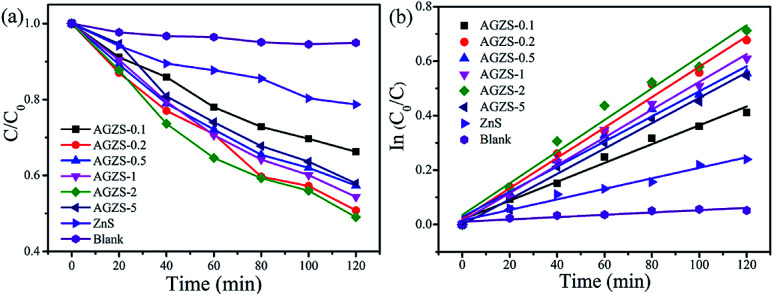
(a) Photocatalytic performance of the ZnS and Ag/ZnS composites in the degradation of MO under UV light; and (b) the kinetic curve.

In order to evaluate the catalytic activity of the composites, MO was used as the degradation target, as shown in Fig. 5a. After introduction of Ag, the Ag/ZnS composites exhibited an improved catalytic activity, which changed regularly with different amounts of Ag. When the mass percentage of Ag to ZnS was two, the composites AGZS-2 exhibited the highest photocatalytic activity, indicating that the amount of Ag plays a key role in obtaining photocatalysts with excellent activity. In addition, the enhanced photocatalytic activity can be attributed to two aspects. On the one hand, the Ag nanoparticles in the composites obtained by NaBH_4_ reduction are tightly bound to ZnS, which is beneficial to the rapid separation of the photogenerated carriers.^41^ On the other hand, the SPR effect of the Ag nanoparticles enhanced the absorption of light, and improved the catalytic performance.

Fig. 5b shows the kinetic results for the ZnS and Ag/ZnS composites. It can be seen that the photocatalytic degradation of MO complies with a quasi-first-order kinetic reaction.^42^ The rate constants of pure ZnS and composite AGZS-2 were 0.00193 and 0.00581 min^−1^, respectively. Compared with pure ZnS, the degradation activity of composite AGZS-2 increased by three times, indicating that the composites exhibit excellent photocatalytic activities. Furthermore, the recycling activity of the Ag/ZnS composites has been tested, and we found that the stability is relatively high (Fig. S2†).

**Fig. 6:**
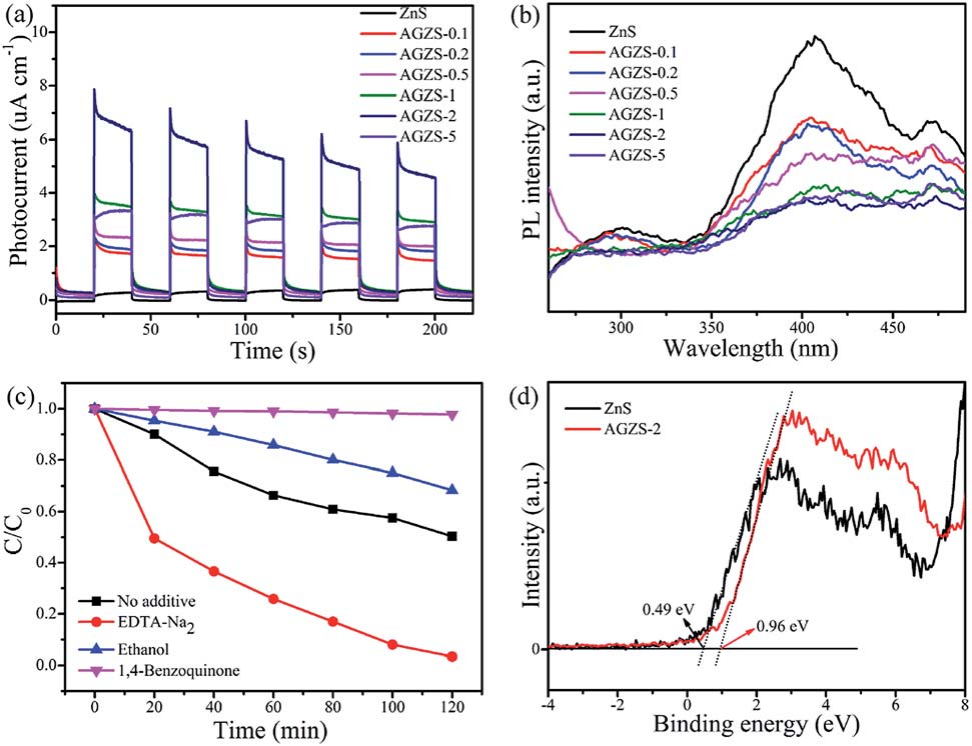
(a) Photocurrent response, (b) PL spectra of pure ZnS and Ag/ZnS composites, (c) species tests for MO degradation by composite AGZS-2 under UV irradiation, and (d) XPS-VB spectrum of composites AGZS-2.

In order to study the photocatalytic mechanism of the Ag/ZnS composites, the photocurrent response, photoluminescence (PL) spectra, capture agents and valence band XPS (XPS-VB) spectra measurements were conducted, as shown in Fig. 6.

From Fig. 6a, it can be seen that the pure ZnS exhibits a relatively low photocurrent response. After the introduction of Ag, the Ag/ZnS composites displayed a significantly increased photocurrent higher than that of ZnS. In particular, the composite AGZS-2 showed the strongest photocurrent signal, indicating that there is an efficient electron transfer between ZnS and Ag. Hence, the introduction of Ag can effectively improve the separation efficiency of the photogenerated electron–holes and exhibit an enhanced activity, which can also be verified by the photocatalytic results (Fig. 5).^43^

Fig. 6b shows the PL spectra of the pure ZnS and Ag/ZnS composites. The PL intensities of the composites significantly decreased after introducing Ag. Among them, the composite AGZS-2 exhibited the lowest PL intensity, indicating that introducing Ag can effectively inhibit the photogenerated charge recombination and further improve the photocatalytic activity.^44^

The role of active components in the catalytic process was investigated using a capture agent experiment. Here, disodium edetate (EDTA-Na_2_), ethanol (ethanol) and *p*-benzoquinone (1,4-benzoquinone) were used as capture agents of holes (h^+^), hydroxyl radicals (·OH) and superoxide radicals (·O_2_^−^), respectively.^45^ The degradation curves of MO after adding the above capture agents are shown in Fig. 6c. When EDTA-Na_2_ was added, the degradation rate to MO by the composite AGZS-2 is obviously improved, indicating that h^+^ is not an active component that affects the degradation process of MO. The degradation efficiency was inhibited to some extent after ethanol was added, indicating that ·OH is one of the active components, but is not the main component. When 1,4-benzoquinone was added to the reaction system, the degradation rate was significantly reduced, indicating that ·O_2_^−^ is the main active component during MO degradation. It can be seen that ·O_2_^−^ is the main active species that affects MO degradation, and ·OH is the second.

The position of the band determines the redox capacity of the photogenerated carriers during the catalytic degradation process. Therefore, XPS-VB spectra measurements of ZnS and composites AGZS-2 were performed, and the results are shown in Fig. 6d. The valence band edge (*E*_VB_) of ZnS and composite AGZS-2 are 0.49 and 0.96 eV, respectively, wherein the potential of the valence band of composite AGZS-2 is higher than that of ZnS (0.49 eV). This indicates that composite AGZS-2 has a stronger oxidizing power and catalytic activity than that of pure ZnS, probably due to the presence of Ag. Combined with the UV-vis results, the *E*_g_ values of the ZnS and composite AGZS-2 are 3.38 and 3.43 eV, respectively. According to the formula *E*_g_ = *E*_VB_ − *E*_CB_, the determined conduction band edge (*E*_CB_) values of the ZnS and composite AGZS-2 are −2.89 and −2.47 eV, respectively.

### 3.3 Characterization and activity of the Au/ZnS composites

**Fig. 7:**
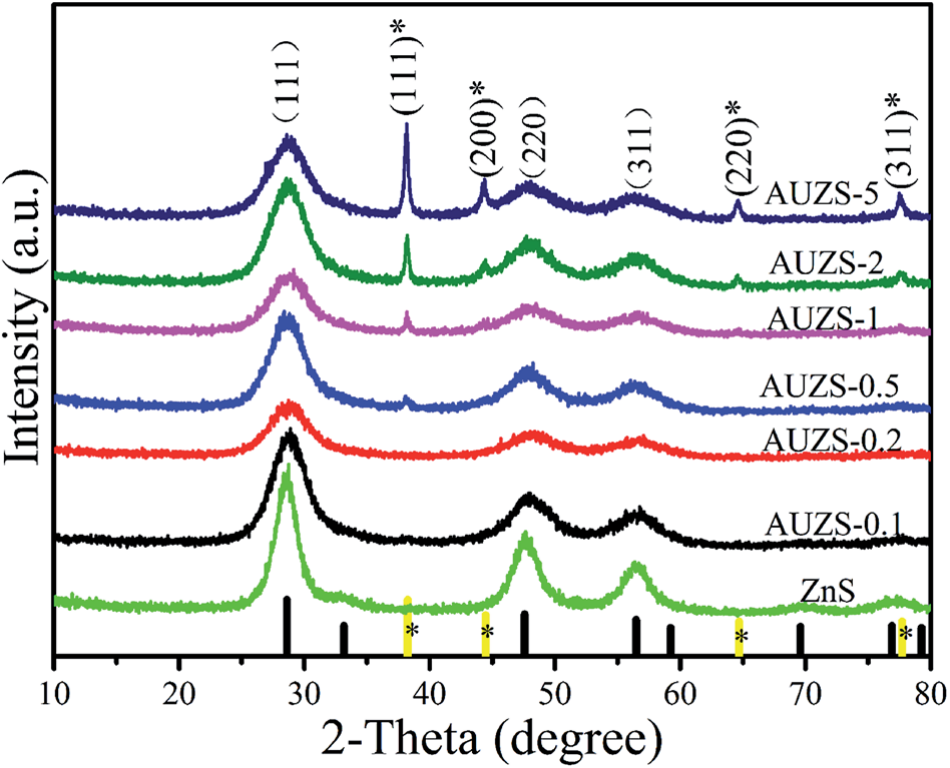
XRD patterns of pure ZnS and Au/ZnS composites (AUZS-0.1–AUZS-5).

In Fig. 7, the XRD patterns of the Au/ZnS composites are basically the same as those of ZnS (JCPDS no. 05-0566). When the mass percentage of Au to ZnS is 0.5, the composite AUZS-0.5 exhibits two diffraction peaks at 38.3° and 44.3°. In comparison with the standard card (JCPDS no. 65-8601), the corresponding diffraction peaks can be designated to the (111) and (200) crystal planes of Au, indicating that the Au/ZnS composites were obtained by solid-phase chemical reaction.^46^ In addition, when the amount of Au increases, the intensity of the diffraction peaks for Au increase, while the intensity of ZnS decreases, possibly due to the change in crystallinity.

**Fig. 8:**
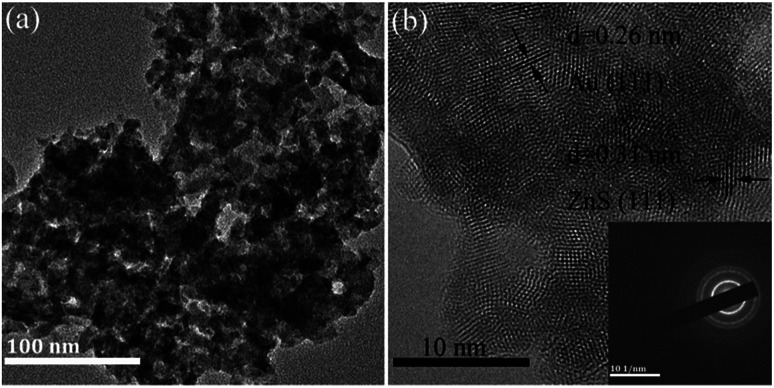
TEM images of the Au/ZnS composite AUZS-2 (a); HRTEM image (b), and SAED patterns (inset).

As for composite AUZS-2, the Au nanoparticles at about 20 nm are distributed on the surface of ZnS (Fig. 8a). While, in Fig. 8b, the crystal plane distances of 0.31 and 0.26 nm correspond to the (111) plane of ZnS and the (111) plane of Au, respectively.^47^ This is in agreement with the XRD results, demonstrating the successful synthesis of the Au/ZnS composites. The SAED pattern in the inset further indicates the polycrystalline properties of the composites.

**Fig. 9:**
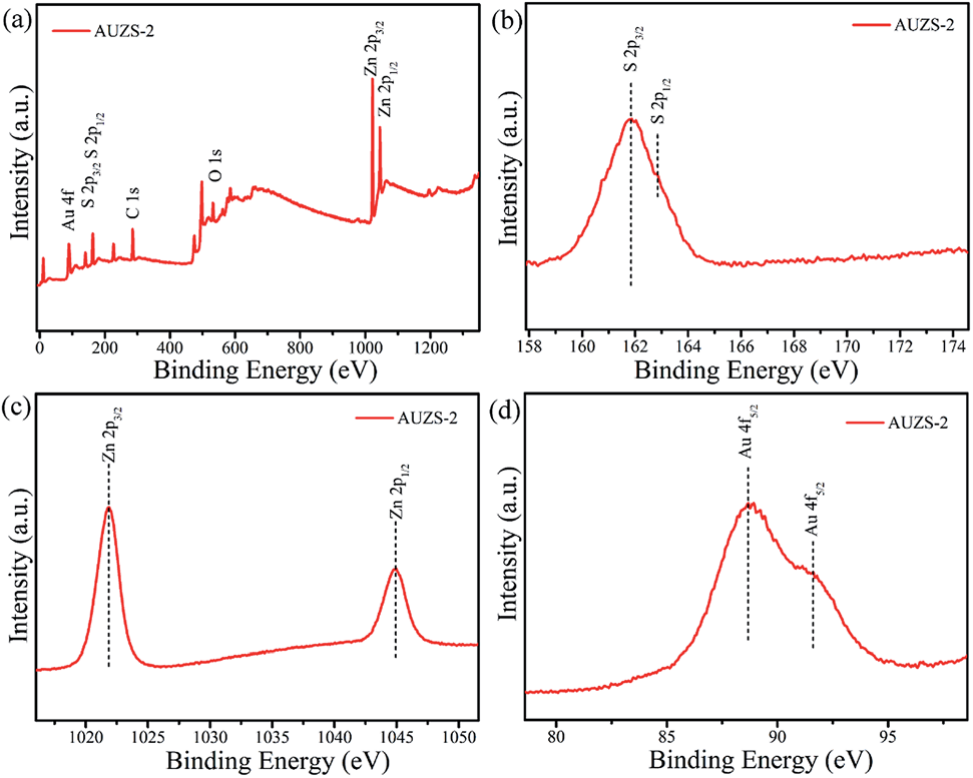
XPS spectra of the Au/ZnS composite AUZS-2: (a) survey; (b) S 2p; (c) Zn 2p; and (d) Au 4f.

The survey spectrum (Fig. 9a) confirmed that there are S, Zn and Au elements in the composite AUZS-2. In Fig. 9b, the characteristic peaks of the S 2p_3/2_ and S 2p_1/2_ orbitals of the composite AUZS-2 are slightly red-shifted to 161.9 and 162.8 eV compared to the pure ZnS, respectively, suggesting there are interactions between Au and ZnS. As for the composite AUZS-2, characteristic peaks for the Zn 2p_3/2_ and Zn 2p_1/2_ orbitals appear at 1021.8 and 1044.8 eV, respectively (Fig. 9c). The two peaks for Au 4f_5/2_ at 88.7 and 91.6 eV are assigned to Au^0^ and Au^1+^, respectively (Fig. 9d), indicating the presence of the Au element in composite AUZS-2.^48^ The above described results show that there are two phases of ZnS and Au in the composites, that is, the Au/ZnS composites are obtained. From the XPS analysis, it can be found that the Au percentage with respect to ZnS was 1.9% in the composite AUZS-2, in agreement with the expected results.

**Fig. 10:**
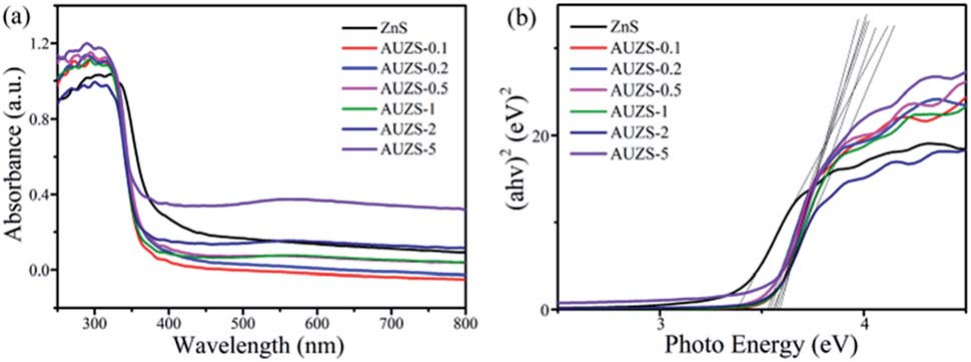
(a) UV-vis spectra; and (b) (*αhν*)^2^*versus hν* of the ZnS and Au/ZnS composites.

After the introduction of Au, the Au/ZnS composites have a significant blue shift compared with that of ZnS (Fig. 10a). At the same time, with the formation of the Au/ZnS composites, a new absorption band appears in the range of 450–800 nm, which is caused by the plasmon resonance effect of Au, indicating the presence of Au in the composites.^49^ According to the Tauc equation *αhν* = *A*(*hν* − *E*_g_)^*n*^, the *E*_g_ of the Au/ZnS composites AUZS-0.1, AUZS-0.2, AUZS-0.5, AUZS-1, AUZS-2, and AUZS-5 are 3.38, 3.57, 3.53, 3.54, 3.57, 3.55 and 3.59 eV, respectively (Fig. 10b). It can be seen that the *E*_g_ of composite AUZS-2 is relatively small, which may be related to the structure and properties.

**Fig. 11:**
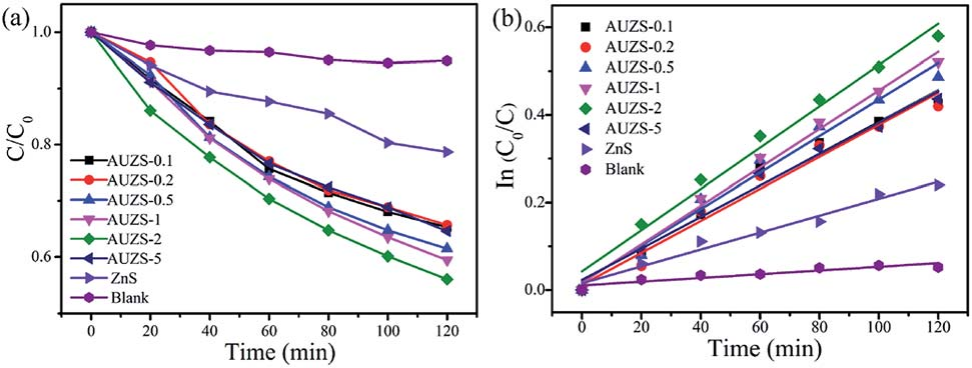
(a) Photocatalytic performance of ZnS and Au/ZnS composites for the degradation of MO under UV light, and (b) the kinetic curve.

The photocatalytic activity of the Au/ZnS composites were also explored as shown in Fig. 11. As the amount of Au increases, the photocatalytic activity of the Au/ZnS composites first increases and then decreases (Fig. 11a). When the mass percentage of Au to ZnS is two, the composite AUZS-2 exhibited the highest photocatalytic performance. The enhancement of the photocatalytic ability can be ascribed to the close combination of Au and ZnS and the SPR effect of Au, which facilitates the rapid separation of photogenerated carriers and thus improves the catalytic performance.^50^

In order to compare the photocatalytic activities of the Au/ZnS composites and Ag/ZnS composites, Fig. S3† was examined. It can be found that 44% of MO was degraded by the Au/ZnS composite AUZS-2 after irradiation for 120 min, lower than that of the Ag/ZnS composite AGZS-2 (49%), indicating that the Ag/ZnS composites possess a higher photocatalytic activity than that of the Au/ZnS composites.

Fig. 11b indicates that the Au/ZnS composite photocatalytic degradation of MO conforms to a quasi-first-order kinetic process. The rate constant of the composite AUZS-2 was 0.00471 min^−1^, three times higher than that of ZnS, indicating that the Au/ZnS composite exhibits an excellent photocatalytic performance after introducing Au. In addition, we can see that the Au/ZnS composites also exhibited a stable photocatalytic activity (Fig. S4†).

**Fig. 12:**
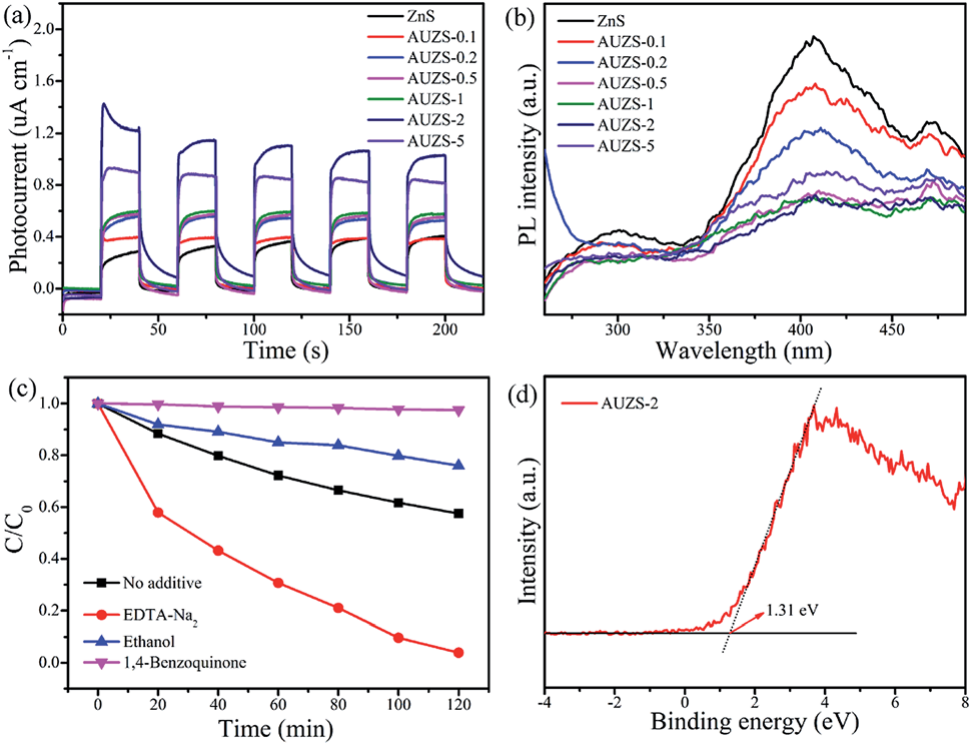
(a) Photocurrent response, (b) PL spectra of the Au/ZnS composites, (c) species tests for degrading MO using composite AUZS-2 under UV irradiation, and (d) XPS-VB spectrum of composite AUZS-2.

Subsequently, the photocatalytic mechanism of the Au/ZnS composites was further studied as shown in Fig. 12. In Fig. 12a, the photocurrent of the Au/ZnS composites increases significantly after introduction of Au, and composite AUZS-2 exhibits the strongest photocurrent signal, indicating that introducing Au can effectively improve the separation efficiency of the photogenerated charges.^51^ In Fig. 12b, the PL intensity of the composites significantly decreased after introducing Au. Wherein the composite AUZS-2 displayed the lowest PL intensity, implying an excellent separation ability of photogenerated charges, which was consistent with the results of the photocurrent analysis (Fig. 12a).^52^ The results of the capture agent experiment (Fig. 12c) indicate that ·O_2_^−^ is the main active substance, followed by ·OH. In addition, a XPS-VB spectrum study was performed on composite AUZS-2, as shown in Fig. 12d. The determined *E*_VB_ of composite AUZS-2 is 1.31 eV, which is higher than that of ZnS (0.56 eV), indicating that composite AUZS-2 has a strong oxidizing ability.^53^ Combined with UV-vis results, the *E*_g_ of the composite AUZS-2 was found to be 3.55 eV. According to the formula *E*_g_ = *E*_VB_ − *E*_CB_, it can be determined that the *E*_CB_ of composite AUZS-2 is −2.24 eV.

### 3.4 Photocatalytic mechanism of the noble metal/ZnS composites

Based on the experimental results of composition, structure, photocatalytic activity, photocurrent, PL spectra, XPS-VB and active species, the reasons for the improvement in the photocatalytic activity of ZnS after introducing noble metals can be explained as follows.

Here, we take the Ag/ZnS composite as an example. First, the improved catalytic activity of the Ag/ZnS composites is mainly due to the rapid generation and migration of photogenerated charges. According to the results of the above mentioned UV-vis and XPS-VB tests, the *E*_CB_ and the *E*_VB_ position of ZnS are −2.89 and 0.49 eV, respectively. According to the literature, the Fermi level of Ag is located at 0.4 eV (*vs.* a normal hydrogen electrode (NHE)).^54^ Under UV irradiation, ZnS is excited and generates electron–hole pairs. Driven by the difference in the ZnS conduction band potential and the Ag Fermi level potential, photogenerated electrons of ZnS will rapidly migrate to the Ag surface, effectively reducing the recombination of the photogenerated electrons and holes on the surface of ZnS, which can also be confirmed by the results of the photocurrent and PL spectra (Fig. 6). However, when the mass percentage of Ag to ZnS increases to five, a new carrier recombination center is generated, thereby reducing the catalytic activity of the Ag/ZnS composites.

**Fig. 13:**
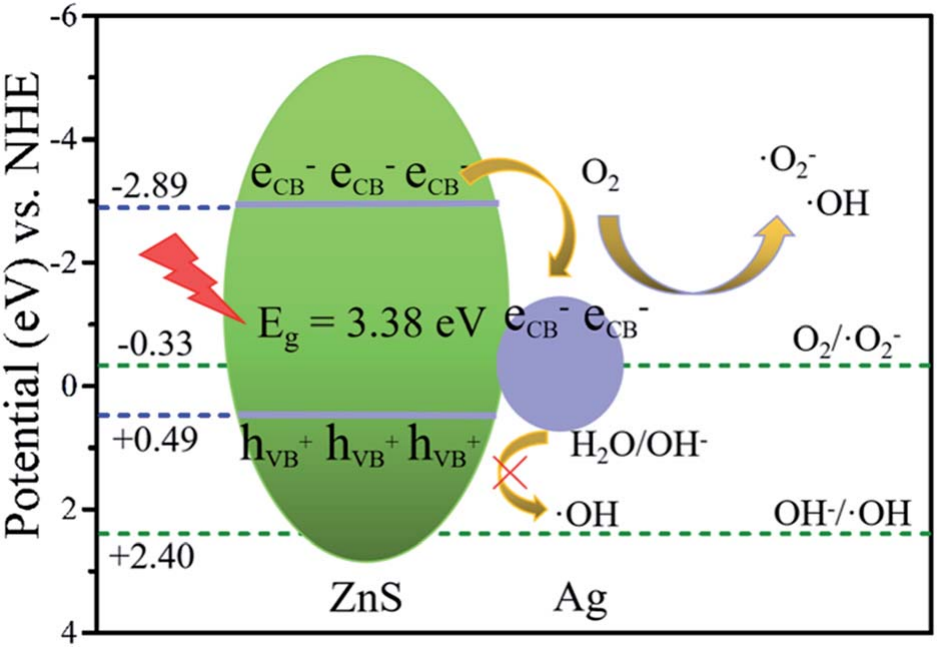
Photocatalytic mechanism for the degradation of MO over the Ag/ZnS composites.

Secondly, the active species involved in the reaction is the direct cause of the enhanced photocatalytic activity of the Ag/ZnS composites. After photogenerated electron–hole separation, it is further converted into an active species (·OH, h^+^, ·O_2_^−^) to complete the degradation of MO. As can be seen from Fig. 13, the valence band position of ZnS (0.99 eV) is higher than the OH^−^/·OH potential (1.99 V *vs.* NHE). Therefore, ·OH (eqn (1)) cannot be obtained by oxidizing OH^−^. However, ·OH appears in the Ag/ZnS composites as shown in Fig. 6c. This may be due to the fact that after introduction of Ag, the reaction time of the electron is increased, and then a multi-electron reaction with oxygen occurs, indirectly generating ·OH (eqn (2)).^55^ In addition, as the photogenerated electrons aggregate on the Ag nanoparticles, the Fermi level increases and is higher than the O_2_/·O_2_^−^ potential (−0.046 V *vs.* NHE), and then the electron can reduce O_2_ to ·O_2_^−^ (eqn (3)). The same conclusion can also be obtained in the degradation of MO by the Au/ZnS composites.

1 OH + h_VB_^+^ →·OH

2 O_2_ + 2H^+^ + 3e_CB_ → OH^−^ +·OH

3 O_2_ + e_CB_ →·O_2_^−^

In summary, the mechanism of the catalytic degradation of MO by noble metal/ZnS composites can be briefly described as follows. Under UV irradiation, photogenerated electrons and holes are generated on the surface of ZnS, and electrons rapidly migrate to noble metal nanoparticles, thereby achieving the effective separation of electrons and holes on the surface of ZnS. Next, electrons on the noble metal nanoparticles react with O_2_ to obtain the active species ·O_2_^−^ and ·OH. Eventually, the active species react with MO and then converts it to H_2_O and CO_2_, thereby achieving the degradation of MO.

## 4. Conclusions

Noble metal/ZnS composites were obtained using a low-temperature solid-phase chemical reaction in the presence of NaBH_4_. UV-vis analysis showed that the noble metal/ZnS composites showed a new absorption band in the range of 450–800 nm, indicating the presence of noble metal in the composites, which is consistent with the XPS results. The noble metal/ZnS composites exhibited enhanced photocatalytic activities compared with pure ZnS. When the mass percentage of the noble metal to ZnS is two, the composites displayed the highest photocatalytic value, suggesting that the content of noble metals has a great influence on the performance. The excellent activities of the noble metal/ZnS composites are mainly due to the rapid migration of carriers and the enhancement of the light absorption capacity, ascribed to the tight combination between ZnS and the noble metal and the SPR effect of the noble metal. The method for obtaining noble metal nanoparticles by NaBH_4_ reduction is simple and rapid, and does not require complicated photoreduction equipment. It is expected to extend the method to the high-volume synthesis of noble metal-based composite photocatalysts.

## Conflicts of interest

There are no conflicts to declare.

## Supplementary Material

RA-010-C9RA07163F-s001
