# Supplementary material for: Facile strategy for the fabrication of noble metal/ZnS composites with enhanced photocatalytic activities
Source: RSC Adv. 2020 Jan 27;10(8):4455–63. doi: 10.1039/c9ra07163f (PMC9048999; doi:10.1039/c9ra07163f)
Supplement: RA-010-C9RA07163F-s001 [file RA-010-C9RA07163F-s001.pdf]

This journal is © The Royal Society of Chemistry 2019

A facile strategy for the fabrication of noble metal/ZnS composites with enhanced photocatalytic activities

Xuekun Jin[a,‡], Fengjuan Chen[a, b,*,‡], Dianzeng Jia[a,*], Yali Cao[a], Haiming Duan[b], Mengqiu Long[c]

[a]Key Laboratory of Energy Materials Chemistry, Ministry of Education, Key Laboratory of Advanced Functional Materials, Autonomous Region, Institute of Applied Chemistry, Xinjiang University, Urumqi, Xinjiang 830046, China.

[b]School of Physics Science and Technology, Xinjiang University, Urumqi 830046, Xinjiang, PR China.

[c]Hunan Key laboratory of Super Micro-structure and Ultrafast Process, Central South University, Changsha 410083, China.

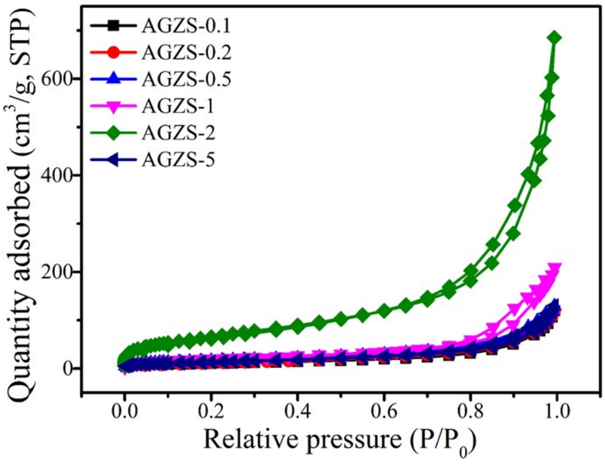

Fig. S1. N_2 adsorption-desorption isotherms for Ag/ZnS composites.

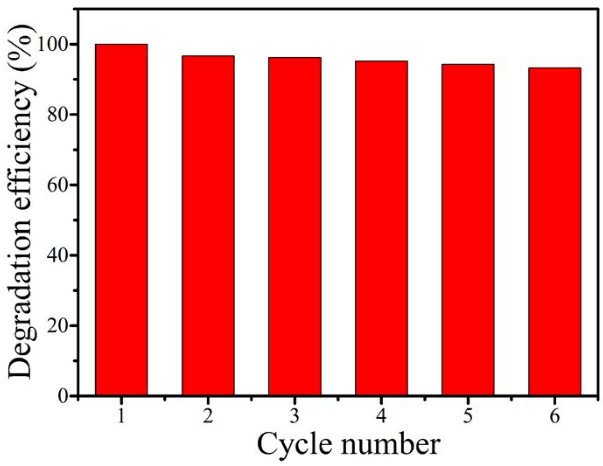

Fig. S2. The recycling activity of Ag/ZnS composites AGZS-2.

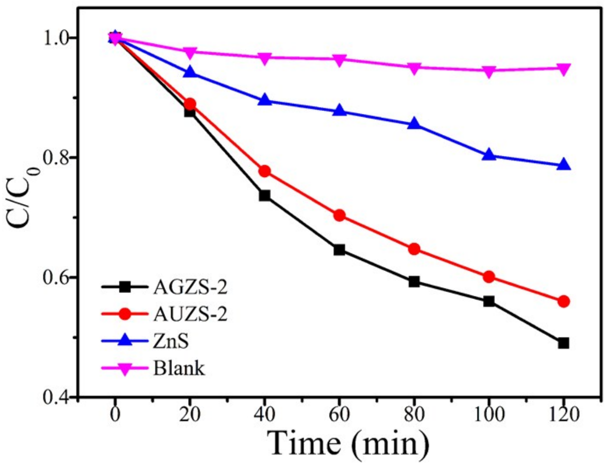

Fig. S3. Comparison photocatalytic activities between Ag/ZnS composites and Au/ZnS composites.

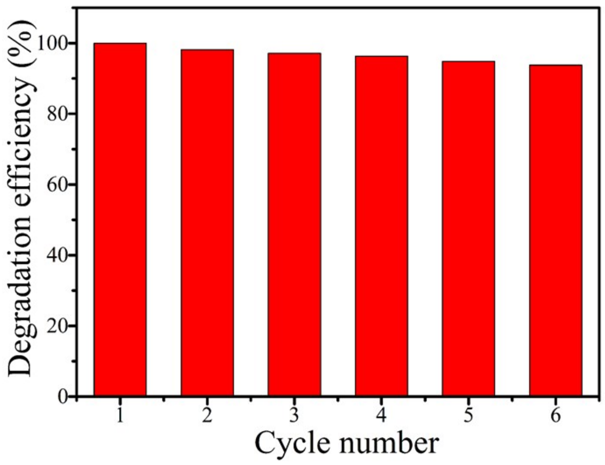

Fig. S4. The recycling activity of Au/ZnS composites AUZS-2.
